# Polymorphisms in *Cyclooxygenase*, *Lipoxygenase*, and *TP53* Genes Predict Colorectal Polyp Risk Reduction by Aspirin in the seAFOod Polyp Prevention Trial

**DOI:** 10.1158/1940-6207.CAPR-23-0111

**Published:** 2023-09-26

**Authors:** John R. Davies, Tracey Mell, Harriett Fuller, Mark Harland, Rasha N.M. Saleh, Amanda D. Race, Colin J. Rees, Louise C. Brown, Paul M. Loadman, Amy Downing, Anne Marie Minihane, Elizabeth A. Williams, Mark A. Hull

**Affiliations:** 1 Leeds Institute of Medical Research, University of Leeds, Leeds, United Kingdom.; 2 Nutrition and Preventive Medicine, Norwich Medical School, University of East Anglia, Norwich, United Kingdom.; 3 Department of Clinical and Chemical Pathology, Faculty of Medicine, Alexandria University, Egypt.; 4 Institute of Cancer Therapeutics, University of Bradford, United Kingdom.; 5 Population Health Science Institute, Newcastle University, United Kingdom.; 6 MRC Clinical Trials Unit at University College, London, United Kingdom.; 7 Norwich Institute of Health Ageing, Norwich, United Kingdom.; 8 Department of Oncology and Metabolism, University of Sheffield, United Kingdom.

## Abstract

Aspirin and eicosapentaenoic acid (EPA) reduce colorectal adenomatous polyp risk and affect synthesis of oxylipins including prostaglandin E_2_. We investigated whether 35 SNPs in oxylipin metabolism genes such as *cyclooxygenase* (*PTGS*) and *lipoxygenase* (A*LOX*), as well as 7 SNPs already associated with colorectal cancer risk reduction by aspirin (e.g., *TP53*; rs104522), modified the effects of aspirin and EPA on colorectal polyp recurrence in the randomized 2 × 2 factorial seAFOod trial. Treatment effects were reported as the incidence rate ratio (IRR) and 95% confidence interval (CI) by stratifying negative binomial and Poisson regression analyses of colorectal polyp risk on SNP genotype. Statistical significance was reported with adjustment for the false discovery rate as the *P* and *q* value. 542 (of 707) trial participants had both genotype and colonoscopy outcome data. Reduction in colorectal polyp risk in aspirin users compared with nonaspirin users was restricted to rs4837960 (*PTGS1*) common homozygotes [IRR, 0.69; 95% confidence interval (CI), 0.53–0.90); *q* = 0.06], rs2745557 (*PTGS2*) compound heterozygote-rare homozygotes [IRR, 0.60 (0.41–0.88); *q* = 0.06], rs7090328 (*ALOX5*) rare homozygotes [IRR 0.27 (0.11–0.64); *q* = 0.05], rs2073438 (*ALOX12*) common homozygotes [IRR, 0.57 (0.41–0.80); *q* = 0.05], and rs104522 (*TP53*) rare homozygotes [IRR, 0.37 (0.17–0.79); *q* = 0.06]. No modification of colorectal polyp risk in EPA users was observed. In conclusion, genetic variants relevant to the proposed mechanism of action on oxylipins are associated with differential colorectal polyp risk reduction by aspirin in individuals who develop multiple colorectal polyps. SNP genotypes should be considered during development of personalized, predictive models of colorectal cancer chemoprevention by aspirin.

**Prevention Relevance:**

Single-nucleotide polymorphisms in genes controlling lipid mediator signaling may modify the colorectal polyp prevention activity of aspirin. Further investigation is required to determine whether testing for genetic variants can be used to target cancer chemoprevention by aspirin to those who will benefit most.

## Introduction

A large body of evidence has accumulated from epidemiologic observations, randomized colorectal polyp prevention trials, and long-term follow-up studies of colorectal cancer outcomes after participation in vascular prevention trials, that regular aspirin use is associated with a 15% to 20% reduction in colorectal cancer risk (1–2). However, aspirin use is not currently recommended for primary or secondary prevention of “sporadic” colorectal cancer due primarily to concerns about the well-recognized gastrointestinal and intracranial bleeding risk associated with aspirin treatment (1). Therefore, a precision approach to primary colorectal cancer chemoprevention is needed to harness the preventative activity of aspirin in those individuals at highest risk of colorectal cancer, while avoiding aspirin use in those most at risk of harm. Several clinical factors are recognized to predict increased colorectal cancer risk (age, male sex, body fatness, family history, previous colorectal polyp history) and aspirin-related bleeding risk (age, hypertension, *Helicobacter pylori* infection; ref. 3).

However, a multivariable risk prediction model utilizing these clinical features has yet to emerge that would support precision chemoprevention largely because of the dearth of an accompanying biomarker(s) of personalized aspirin efficacy that could pinpoint individuals who are either sensitive to, or resistant to, the anti–colorectal cancer activity of aspirin (3). There has been much interest in the stable urinary prostaglandin (PG) E_2_ metabolite, PGE-M, as a predictive risk, or therapeutic response, biomarker of colorectal polyp prevention by aspirin (4), although it seems unlikely that a threshold pretreatment or on-treatment urinary PGE-M level will have suitable test performance characteristics to enhance a clinically useful risk model (4).

An alternative approach has been to identify genetic variants that may be linked to differential risk reduction associated with aspirin or other nonsteroidal anti-inflammatory drug (NSAID) use (5). Investigation of SNPs in genes believed to be relevant to the mechanism of action of aspirin [*Prostaglandin G/H Synthase* (*PTGS*); also known as *Cyclooxygenase* (COX)-1 and -2] in *post hoc* analyses of randomized colorectal polyp prevention trials of aspirin has demonstrated a possible interaction between aspirin and *PTGS2* SNP rs4648310 (6). However, there were null associations for *PTGS2* SNPs rs20417, rs2745557, rs5277, rs5275, and rs20432, as well as *PTGS1* SNP rs3842787, in those randomized trial datasets (6–7).

COX-1 and COX-2 enzymes are direct acetylation targets of aspirin, leading to inhibition of downstream synthesis of pro-tumorigenic PGE_2_ from the omega-6 polyunsaturated fatty acid (PUFA) COX substrate C20:4*n*-6 arachidonic acid (AA; ref.^8^). Alternatively, aspirin inhibition of COX activity leads to substrate diversion of AA toward lipoxygenase (LOX; encoded by *ALOX* genes)-mediated synthesis of other oxylipins with antitumorigenic activity such as leukotriene (LT) B_4_ (9).

Moreover, expression of 15-PG dehydrogenase (encoded by *HPGD*), the enzyme responsible for inactivating PGE_2_, has been shown to predict colorectal cancer risk reduction by aspirin (10).

Despite the relevance of other genes controlling oxylipin synthesis for the chemopreventative property of aspirin, the relationship between SNP genotypes in a wide range of genes controlling oxylipin synthesis and levels (including *PTGS1* and *PTGS2*, as well as *ALOX5*, *ALOX12*, and *HPGD*) and the effect of aspirin on colorectal polyp risk has not been addressed previously in a randomized trial setting.

The omega-3 PUFA C20:5*n*-3 eicosapentaenoic acid (EPA) also has colorectal cancer chemoprevention activity (11). It is an alternative substrate for multiple monooxygenases including the COX isoforms and the LOX family of enzymes (12). EPA is an efficient COX-1 inhibitor and metabolism by COX (and LOX isoforms) leads to synthesis of 3- and 5-series oxylipins such as PGE_3_ and LTB_5_, which have reduced proinflammatory and protumorigenic activity compared with their 2- and 4-series counterparts, perhaps contributing to the anti–colorectal cancer activity of EPA (12–14). Therefore, the same genes controlling oxylipin synthesis and levels that could be relevant to aspirin chemoprevention could also modify anti–colorectal cancer activity of EPA.

Several other SNPs that are not directly related to oxylipin metabolism have also been demonstrated to modify colorectal cancer risk reduction associated with aspirin, for example rs6983267 (15) and rs2965667 (5), as well as being linked to increased colorectal cancer risk in other observational studies (rs1042522; ref. 16).

The seAFOod polyp prevention trial was a 2×2 factorial, randomized, placebo-controlled trial of aspirin 300 mg daily and EPA 2,000 mg free fatty acid equivalents daily, alone or in combination, for 12 months in 707 individuals aged 55–73 years with “high-risk” colorectal polyp findings (≥3 polyps, if one polyp was ≥10 mm; or ≥5 polyps of any size) at screening colonoscopy in the English Bowel Cancer Screening Programme (BCSP; refs. 17, 18). Trial participants mirrored individuals undergoing screening colonoscopy after a positive fecal occult blood test (FOBt) in the BCSP with a male predominance and high prevalence of excess body weight (17, 18). The primary “at the margins” analysis compared aspirin and EPA separately against their respective placebos, assuming no interaction between the two interventions (17–18). While the seAFOod trial found no evidence for an effect of aspirin or EPA on the primary outcome of adenoma detection rate (the % of participants with one or more colorectal polyps), aspirin use was associated with a significant reduction in total colorectal polyp risk [measured as mean total polyp (including adenomatous and serrated polyps) number per participant] one year after clearance colonoscopy compared with placebo treatment (17, 18). In contrast, EPA use was not associated with reduced total colorectal polyp risk but was associated with a statistically significant reduction in risk of left-sided (distal to the splenic flexure), adenomatous polyps (17, 18).

We tested the relationship between SNPs in genes controlling oxylipin synthesis and levels, which are relevant for the putative mechanism(s) of the anticancer activity of aspirin and EPA, as well as SNPs in selected genes already linked to modification of colorectal cancer risk reduction by aspirin, and colorectal polyp outcomes in the seAFOod polyp prevention trial.

## Materials and Methods

### The seAFOod polyp prevention trial biobank

This study is part of a wider program of investigations using the seAFOod trial biobank and post-trial BCSP colonoscopy outcomes called STOP-ADENOMA (ISRCTN05926847). The study was conducted in accordance with the Declaration of Helsinki. Ethical approval for this study was granted by London and Surrey Borders Research Ethics Committee (19/LO/1655). The seAFOod Trial biobank has been described elsewhere (17). Blood samples were obtained, and buffy coat leukocytes were collected and stored locally at −20°C and in the central trial Biobank at −80°C, as described in the Trial Laboratory Manual and detailed Trial report (18, 19).

### DNA extraction

DNA was extracted from a single buffy coat extract per seAFOod trial participant using an in-house phenol-chloroform method, followed by washing with absolute ethanol and storage at 4°C in DNase/RNase-free water, prior to spectrophotometric assessment of the DNA quantity and quality. If any sample had a DNA yield <20 ng/μL, a second sample for that individual was obtained from the Biobank for DNA extraction.

### SNP genotyping

Genotyping was carried out using the Fluidigm microfluidic SNP genotyping system (Fluidigm), using custom-built SNP Type genotyping assays (Fluidigm) for 92 SNPs (Supplementary Table S1).

A multiplexed preamplification PCR was carried out to increase the available template DNA and reduce allelic drop-out associated with low template concentration. DNA samples were run on 96.96 Dynamic Array IFCs (integrated fluidic circuits), which were primed and loaded using the IFC Controller HX (96.96 arrays), as per manufacturer's instructions. In total, 691 individual samples including 10 technical duplicates, 31 participant duplicate samples and 1 participant triplicate sample, were analyzed, representing 648 individual participant DNA samples.

Genotyping PCR and measurement of endpoint fluorescent values were carried out using the Fluidigm BioMark HD system. Data were analyzed and genotyping calls made using Fluidigm SNP Genotype Analysis software.

Fourteen SNPs failed quality control; one SNP was monoallelic, one SNP was repeatedly called differently in duplicate sample runs, and 12 SNPs had an absence of genotype call >5% (Supplementary Table S1).

Therefore, genotype data for 78 SNPs were available for analysis inside the seAFOod trial database. Twelve SNPs did not satisfy Hardy–Weinberg equilibrium (*P* < 0.05 with Benjamini–Hochberg correction for multiple testing). However, on inspection of scatter plots produced during Fluidigm genotyping, “true calls” did not overlap with “fails” suggesting that this was not caused by sampling error. Therefore, these SNPs were included in subsequent analyses to avoid missing a relationship with clinical outcomes given that these SNPs could be causally related to colorectal polyp development (and thus seAFOod trial recruitment) explaining absence of Hardy–Weinberg equilibrium.

This report is restricted to an analysis of the relationship between the 35 SNPs in genes controlling oxylipin synthesis and degradation (*PTGS1*; 7 SNPs: *PTGS2*; 5 SNPs: *ALOX5*; 8 SNPs: *ALOX12*; 10 SNPs: *ALOX15*; 3 SNPs: *HPGD*; 2 SNPs), as well as 7 SNPs previously associated with differential colorectal cancer risk reduction in aspirin users (5, 15–16), and colorectal polyp risk in the seAFOod polyp prevention trial. Genetic variants of interest in *PTGS* and *ALOX* genes were identified as part of the European Union-Biotechnology and Biological Sciences Research Council (UK)-funded Fatty Acid Metabolism (FAME) Consortium - Interlinking Diet with Cardio-metabolic Health. Briefly, all SNPs in *PTGS* and *ALOX* genes were grouped into linkage disequilibrium (LD) blocks using PLINK v1.9 software (*R*^2^ ≥ 0.8, minor allele frequency ≥ 5%; ref. 20). A tagging SNP for each LD block was selected on the basis of prior disease association (from GWAS and pharmacogenetics databases), pathogenicity and position (within promotors, exons, or splice sites).

Data on the relationship between the SNPs in genes controlling PUFA metabolism (Supplementary Table S1) with blood and rectal mucosal levels of PUFAs measured in the seAFOod polyp prevention trial, as well as colorectal polyp risk, are the subject of a separate report.

### Statistical analysis

Linkage disequilibrium between SNPs in individual genes was analyzed using the LDmatrix tool (NIH) to derive *R*^2^ values for paired SNP relationships.

Only seAFOod trial participants, for whom there were trial colonoscopy outcome data, were included in this SNP analysis. Baseline characteristics [age, sex, body mass index (BMI), diabetes, tobacco smoking status, alcohol intake] of the seAFOod trial participants included in the SNP analysis were analyzed using the *χ*^2^ test or Mann–Whitney *U* test, as appropriate, to confirm similarity with the original seAFOod trial cohort and ensure continuing balance across the trial treatment allocations.

Interactions of SNP genotypes with trial interventions were analyzed “at the margins” (i.e. active aspirin vs. placebo aspirin, and active EPA vs. placebo EPA, regardless of the other intervention in the 2×2 factorial trial), consistent with the primary seAFOod trial analysis (that assumed no treatment interaction; refs. 17, 18).

We analyzed total colorectal (combined adenomatous and serrated hyperplastic) polyp number per participant (previously termed adenoma per participant in the original seAFOod trial analysis; refs. 17, 18) in keeping with current colorectal polyp classification (21). We also stipulated analysis of adenomatous polyp number per participant given that a secondary outcome of the seAFOod trial was that EPA (but also aspirin) specifically reduced risk of adenomatous polyps (17–18). Descriptive data were tabulated for the possible genotypes for each SNP across the trial intervention comparisons (active aspirin vs. placebo aspirin, and active EPA vs. placebo EPA) with univariate statistical testing for each genotype by the Kruskal–Wallis rank test. Distribution dot plots were also drawn to compare the colorectal polyp count distribution across genotypes for each SNP.

All SNPs were then used to stratify a negative binomial regression model of colorectal polyp number (justified by a positive skewed distribution of individual colorectal polyp number values in the seAFOod trial; ref. 18), which was adjusted for the hospital site where the colonoscopy occurred, and for whether a repeat colonoscopy (for example, for a polypectomy site check) had taken place at baseline, to aid comparison with the effect sizes for aspirin and EPA reported in the primary seAFOod trial analysis (17, 18). Data are reported as the incidence rate ratio (IRR) and 95% confidence interval (CI). For SNPs that had 40 or more rare homozygotes, the models were stratified at three levels (common homozygote, heterozygote, rare homozygote). In all other cases with fewer cases than the arbitrary threshold of 40 rare homozygotes, heterozygotes, and rare homozygotes were collapsed to ensure a sufficient number of cases in the model (22). For models that showed a statistically significant relationship (*P* ≤ 0.05) between colorectal polyp number and intervention for some strata, an interaction test between the SNP and intervention was conducted fitting the negative binomial regression model with additional terms for interaction between the treatment and SNP variables.

Each analysis was repeated using a Poisson regression model, with the same adjustments, to mirror the seAFOod trial analysis (17–18).

Statistical significance was specified as *P* < 0.05. Given the relatively large number of individual analyses for SNP × colorectal polyp number interactions, as well as two interventions (aspirin and EPA) and two colorectal polyp outcomes, the positive false discovery rate (pFDR) was described by the *q* value for each of the models including individual SNPs (23).

All statistical analyses were conducted using Stata version 17.0.

### Data availability

The data generated in this study are available upon request from the corresponding author and with approval from the study Sponsor (University of Leeds, Leeds, United Kingdom).

## Results

### seAFOod trial participant samples

**Figure 1.**
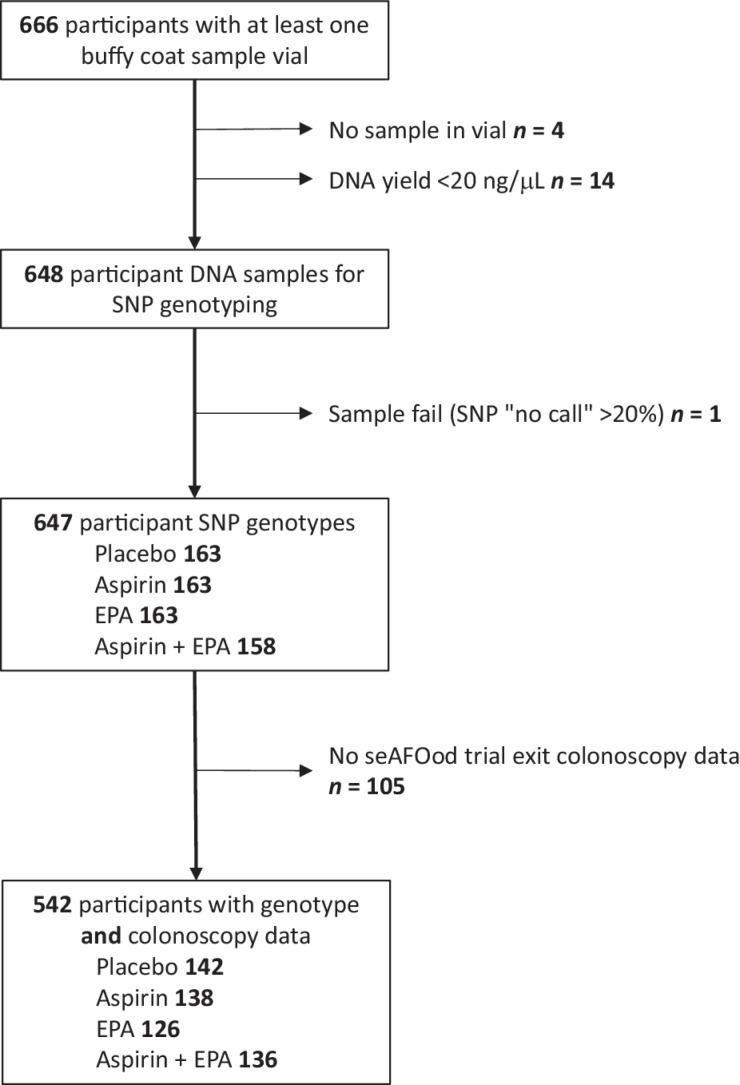
seAFOod trial participants and samples contributing to the gene × treatment interaction analysis. 666 of 707 seAFOod trial participants provided at least one buffy coat sample for DNA extraction. The number of samples lost during either DNA extraction or SNP genotyping is described at each stage. The final study population consisted of 542 participants for whom SNP genotype and trial exit colonoscopy data were available. EPA, eicosapentaenoic acid; SNP, single-nucleotide polymorphism.

A total of 666 trial participants had at least one buffy coat sample vial in the seAFOod trial biobank (Fig. 1). There was no material in a single cryovial in four cases and the DNA yield was below 20 ng/mL in 14 cases despite DNA extraction from a second sample vial, leaving 648 individual participant DNA samples for SNP genotyping. Following characterization by Fluidigm 96.96 IFC assay, one DNA sample yielded a SNP “no call” rate of >20% and was excluded, leaving 647 individual participant SNP genotype profiles for analysis.

A total of 542 seAFOod trial participants, for whom SNP genotype data were available, also had colonoscopy outcome data from the seAFOod trial. Reasons for lack of primary (colorectal polyp) outcome data for some trial participants are detailed in the seAFOod trial CONSORT diagram (17). The clinical characteristics of this study cohort are detailed in Table 1. The clinical features of the cohort used for the SNP analysis were not significantly different from the original randomized trial population (Table 1; ref. 17). There was also no significant difference in the clinical characteristics of the trial participants contributing to this genetic analysis according to treatment allocation to aspirin or EPA versus their respective placebos (Table 1).

**Table 1.** Clinical characteristics of the study cohort of 542 seAFOod trial participants who had a SNP genotype profile and trial colonoscopy outcome data.

	Placebos only	Aspirin only	EPA only	Aspirin & EPA	*P* for aspirin vs. no aspirin comparison	*P* for EPA vs. no EPA comparison	*P* for comparison of study cohort with *n* = 165 excluded trial participants
Number	142	138	126	136			
Age [median (IQR)]	64.5 (7.9)	64.9 (6.2)	65.1 (6.2)	66.4 (7.7)	0.22	0.29	0.42
Sex [male: female (% female)]	113:29 (20.4)	109:29 (21.0)	100:26 (20.6)	114:22 (16.2)	0.48	0.58	0.33
Body mass index (%)^a^					1.0	0.50	0.90
Normal (<24.9 Kg/m^2^)	23 (16.2)	20 (14.5)	23 (18.3)	30 (22.1)			
Overweight (25.0–29.9 Kg/m^2^)	64 (45.1)	60 (43.5)	51 (40.5)	60 (44.1)			
Obese (≥30.0 Kg/m^2^)	54 (38.0)	58 (42.0)	50 (40.0)	46 (33.8)			
Diabetes [*n* (%)]	20 (14.1)	14 (10.1)	13 (11.5)	10 (7.4)	0.18	0.20	0.16
Tobacco smoking					0.35	0.50	0.16
Never	52 (36.6)	51 (37.0)	49 (38.9)	53 (39.0)			
Ex-smoker	65 (45.8)	66 (47.8)	68 (54.0)	58 (42.6)			
Current	25 (17.6)	21 (15.2)	9 (7.1)	25 (18.4)			
Alcohol units per week (median; IQR)	3 (1)	2 (1)	2 (1)	3 (1)	1.0	0.93	0.92

^a^Missing body mass index data for one case in the placebos only group and two cases in the EPA group.

### Relationship between individual SNPs and colorectal polyp number according to seAFOod trial treatment allocation

#### PTGS1, PTGS2, and HPGD

Comparison of total colorectal polyp number per participant between those allocated aspirin versus placebo according to genotype for each *PTGS1*, *PTGS2*, and *HPGD* SNP revealed that *PTGS1* rs4837960 common homozygotes (G:G) and *PTGS2* rs2745557 compound heterozygotes (G:A)-rare homozygotes (A:A) were associated with a statistically significant reduction in total colorectal polyp number in aspirin users versus nonaspirin users in contrast to the other genotype (Supplementary Fig. S1; Supplementary Table S2). All the other *PTGS1*, *PTGS2* and *HPGD* SNPs did not reach statistical significance for a difference in total colorectal polyp number between aspirin and placebo users according to genotype (Supplementary Table S2). For *PTGS1* rs4837960, aspirin use was associated with a reduction in total colorectal polyp risk in homozygotes for the major allele [IRR, 0.69 (0.53–0.90); *P* = 0.006; pFDR *q* = 0.06], but not in individuals with one or more minor T alleles [IRR, 0.91 (0.59–1.40); *P* = 0.7; Table 2]. However, the *P*_int_ value for this SNP failed to reach the prespecified threshold for statistical significance (*P*_int_ = 0.3 for TT+GT vs. GG; Table 2). A similar IRR value for rs4837960 was obtained in a Poisson model and using adenomatous polyp number per participant as the outcome. For the *PTGS2* SNP rs2745557, total colorectal polyp risk reduction was restricted to individuals with one or more minor (A) alleles [IRR, 0.60 (0.41–0.88); *P* = 0.009; pFDR *q* = 0.06], but not common homozygotes (*P*_int_ = 0.2; Table 2). A similar IRR value for rs2745557 was also obtained for this *PTGS2* SNP in a Poisson model and using adenomatous polyp number per participant as the outcome.

**Table 2.** SNP genotypes in *PTGS* genes that are associated with modification of the effect of aspirin on colorectal polyp number in the seAFOod polyp prevention trial.

				Common homozygote				Heterozygote + rare Homozygote				
SNP ID	Gene	Major allele	Minor allele	*n*	IRR (95% CI)	*P*	*q*	*n*	IRR (95% CI)	*P*	*q*	*P*_interaction_
rs4837960	*PTGS1*	G	T	397	0.69 (0.53–0.90)	0.006	0.06	136 + 9	0.91 (0.59–1.40)	0.7	0.8	0.3 (TT+ GT vs. GG)
rs2745557	*PTGS2*	G	A	347	0.83 (0.62–1.09)	0.2	0.3	178 + 17	0.60 (0.41–0.88)	0.009	0.06	0.2 (AA +GA vs. GG)

Note: The IRR and 95% CI for colorectal polyp number is for aspirin *versus* no aspirin in a negative binomial regression model.

None of the *PTGS1*, *PTGS2*, or *HPGD* SNP genotypes, including rs4837960 and rs2745557, reached univariate statistical significance for a difference in total colorectal polyp number or adenomatous polyp number between individuals who received either active EPA or placebo EPA (Supplementary Fig. S1).

#### ALOX5 and ALOX12

Univariate analysis of individual SNPs and total colorectal polyp number in aspirin and EPA users compared with individuals who were allocated to the respective placebo intervention demonstrated statistical significance for the *ALOX5* SNP rs7090328 and for three *ALOX12* SNPs (rs2073438, rs2920421, and rs2271316, which were in strong LD - *R*^2^ between 0.671 and 0.871 for all pairwise comparisons), for aspirin users (Supplementary Fig. S2; Supplementary Table S2). In the negative binomial regression model, *ALOX5* rs7090328 was associated with modification of the effect of aspirin, with a reduction in total colorectal polyp risk in homozygotes for the minor allele [IRR, 0.27 (0.11–0.64); *P* = 0.003; pFDR *q* = 0.05], but not in individuals with one or more major A alleles [IRR (AA), 0.81 (0.59–1.11); *P* = 0.2], with a *P*_int_ value of 0.03 (Table 3). A similar IRR value for rs7090328 related to aspirin use was obtained in a Poisson model and using conventional adenomatous polyp number per participant as the outcome. For the *ALOX12* SNPs, total colorectal polyp risk reduction by aspirin was restricted to common homozygotes, but not those with one or more minor alleles (*P*_int_ = 0.02 for rs2073438 and rs2920421, and *P*_int_ = 0.06 for rs2271316; Table 3). Again, similar IRR values were also obtained for the *ALOX12* SNPs in a Poisson model and using adenomatous polyp number per participant as the outcome.

**Table 3.** SNP genotypes in *ALOX* genes and the *TP53* gene that are associated with modification of the effect of aspirin on colorectal polyp number in the seAFOod polyp prevention trial.

				Common homozygote				Heterozygote				Rare homozygote				
SNP ID	Gene	Major allele	Minor allele	*n*	IRR (95% CI)	*P*	*q*	*n*	IRR (95% CI)	*P*	*q*	*n*	IRR (95% CI)	*P*	*q*	*P*interaction
rs7090328	*ALOX5*	G	A	284	0.81 (0.59–1.11)	0.2	0.3	217	0.76 (0.54–1.09)	0.1	0.3	39^a^	0.27 (0.11–0.64)	0.003	0.05	0.8 (AG *v* GG)
																0.03 (AA *v* GG)
rs2073438	*ALOX12*	G	A	236	0.57 (0.41–0.80)	0.001	0.05	239	1.00 (0.71–1.41)	1.0	1.0	56	0.90 (0.45–1.83)	0.8	0.9	0.02 (AG *v* GG)
																0.3 (AA *v* GG)
rs2920421	*ALOX12*	G	A	220	0.55 (0.39–0.78)	0.001	0.05	254	0.96 (0.69–1.34)	0.8	0.9	68	0.86 (0.45–1.64)	0.7	0.8	0.02 (AG *v* GG)
																0.3 (AA *v* GG)
rs2271316	*ALOX12*	G	C	175	0.56 (0.38–0.82)	0.003	0.05	269	0.91 (0.66–1.26)	0.6	0.8	96	0.86 (0.50–1.49)	0.6	0.8	0.06 (CG *v* GG)
																0.2 (CC *v* GG)
rs1042522	*TP53*	G	C	290	0.80 (0.59–1.09)	0.2	0.3	205	0.82 (0.57–1.18)	0.3	0.5	45	0.37 (0.17–0.79)	0.01	0.06	0.9 (CG *v* GG)
																0.06 (CC *v* GG)

Note: The IRR and 95% CI for colorectal polyp number is for aspirin versus no aspirin in a negative binomial regression model.

^a^Rare homozygotes for rs7090328 were *n* = 40 but missing data caused one case to drop out of the regression model.

None of the *ALOX* SNP genotypes reached univariate statistical significance for a difference in total colorectal polyp number or adenomatous polyp number between individuals who received either active EPA or placebo EPA (Supplementary Fig. S2).

#### SNPs associated with differential colorectal cancer risk reduction in aspirin users, as well as with overall colorectal cancer risk

The only SNP in the panel of polymorphisms previously linked to modification of colorectal cancer risk (5, 15, 16), which demonstrated statistical significance for modification of the association of aspirin use and total colorectal polyp number, was rs104522, which is a SNP in the *TP53* tumor suppressor gene (Supplementary Fig. S3; Supplementary Table S2). In the negative binomial regression model, colorectal polyp risk reduction by aspirin was significant for rare homozygotes [IRR, 0.37 (0.17–0.79); *P* = 0.01; pFDR *q* = 0.06] with a *P*_int_ = 0.06, but not for individuals with one or more major alleles (Table 3). Similar results were obtained in the Poisson model and using adenomatous polyp number per participant as the outcome.

*TP53* rs104522 genotype was not associated with any differential effect of EPA on total colorectal polyp number or adenomatous polyp number (Supplementary Fig. S3).

## Discussion

Using a panel of SNPs in genes controlling oxylipin signaling relevant to colorectal carcinogenesis and the pharmacology of aspirin and EPA, we report the association of several SNPs in genes encoding COX-1 (*PTGS1*), COX-2 (*PTGS2*), 5-LOX (*ALOX5*), and 12-LOX (*ALOX12*) with differential polyp prevention efficacy of aspirin, but not EPA, in the seAFOod trial.

The association of *PTGS1* SNP rs4837960 and *PTGS2* SNP rs2745557 with colorectal polyp risk reduction by aspirin has not been reported previously. We note that the *P*_int_ and pFDR values for these SNPs did not reach the prespecified level for significance and the results require independent verification. The *PTGS1* SNP rs4837960 is in LD (*R*^2^ = 0.93) with another *PTGS1* SNP rs3842787, which has been reported to interact with NSAID use for cancer risk (24), although null findings were reported for this SNP in the UKCAP colorectal polyp prevention trial (7). Both rs4837960 and rs2745557 are intronic and the possible functional consequences of homozygosity for the major G allele at rs4837960, or presence of at least one minor allele (A) at rs2745557, are not known.

A previous analysis of the interaction between *PTGS2* SNPs and aspirin use related to colorectal polyp recurrence in the Aspirin/Folate Polyp Prevention Study (AFPPS) described a possible interaction between rs4648310 and aspirin use (81 mg daily) (6). SNP rs4648310 is 8.7K base pairs downstream from rs2745557 and so is likely to have an association with aspirin efficacy independent of rs2745557 genotype.

We did not detect an interaction between the *PTGS2* promoter SNP rs20417 (-765G>C) and aspirin or EPA use in seAFOod trial participants. rs20417 has been reported to approach statistical significance (*P* = 0.07) for an interaction with NSAID use for reduction in colorectal adenomatous polyp risk in one case–control study (494 cases and 584 controls), but not in another smaller study (25). There was also no interaction between rs20417 and aspirin use in the UKCAP aspirin polyp prevention trial (7). Our data support the contention that there is no interaction between rs20417 and aspirin use for colorectal polyp reduction. It should be noted that the AFPPS and UKCAP trial genotype studies used the “adenoma detection rate” (the % number of individuals with one or more colorectal polyps at follow-up colonoscopy) as a measure of polyp recurrence risk (6–7), whereas we report the association of genotype with reduction in colorectal polyp number in the seAFOod trial (noting that the “adenoma detection rate” was the null primary outcome of the seAFOod trial (17–18)). It will be important, if possible, to perform meta-analysis of the aspirin polyp prevention trials that include genotype data, in order to harmonize colorectal outcome genotype–phenotype correlations (AFPPS and UKCAP studies collected colorectal polyp number as a secondary outcome).

One of the *ALOX12* SNPs that was associated with differential colorectal polyp prevention activity of aspirin in our study (the intronic SNP rs2920421) has been reported to interact with NSAID use in a case–control study of colorectal cancer risk (26). In that study, NSAID use was associated with decreased colorectal cancer risk in heterozygous rs2920421 genotypes, but not major or minor homozygotes (26). In a separate case–control study, rs2073438 (homozygotes for the minor A allele), which is in strong LD with rs2920421, was associated with reduced rectal cancer risk, but not colorectal polyp risk or an interaction with NSAID use (27). Consistent with biological relevance of the *ALOX5* and *ALOX12* SNPs found to be associated with differential colorectal polyp risk in aspirin users in the seAFOod trial, these SNPs were all associated with expression quantitative trait loci for *ALOX5* and *ALOX12* in the sigmoid and transverse colon in the Genotype-Tissue Expression Project (GTEx) database.

We also demonstrated that homozygosity for the SNP (rs104522; G>C; Arg72Pro) in the coding region of the TP53 tumor suppressor gene was associated with colorectal polyp reduction in aspirin users in the seAFOod trial, in contrast with the group of individuals that had at least one major (G) allele, who did not demonstrate a reduction in colorectal polyp risk associated with aspirin use. At least one C allele at rs104522 has been reported to have an OR of 1.16 (1.05, 1.30) for presence of one or more colorectal adenomas compared with homozygous G:G individuals in a meta-analysis of genetic association studies of colorectal adenomas (28). The role of p53 in the proapoptotic activity of aspirin and other nonsteroidal anti-inflammatory drugs remains unclear (29–30). However, it is plausible that the alteration in p53 function associated with rs104522 could alter aspirin chemopreventive activity (31). The relationship between rs104522 and colorectal polyp prevention activity of aspirin requires validation in an independent study, which would ideally have sufficient power to distinguish between the two colorectal polyp types (adenomatous and serrated) that have different molecular pathogenesis (32).

We did not observe any gene–supplement interaction for any SNP related to the modest effect of EPA use on colorectal polyp number in the seAFOod trial. The reduction in total colorectal polyp number associated with EPA use in the seAFOod trial was modest and just failed to reach statistical significance (17–18). However, we also tested the SNPs for modification of the effect of reduction in colorectal adenoma number by EPA that was a statistically significant finding from the seAFOod trial (17–18), which generated null findings. There has been no previous study of potential genetic modifiers of the effect of omega-3 PUFAs on colorectal cancer risk. However, COX-2 SNPs rs5275 and rs4648310 have been reported to modify the association between dietary omega-3 PUFA intake and prostate cancer risk (33–34).

Strengths of this study include the comprehensive coverage of the seAFOod trial population whereby 77% of 707 seAFOod trial participants had both colonoscopy and genotype data available. We used a relatively small, custom-built SNP array of relevant genes based on *a priori* knowledge of aspirin and EPA anticancer pharmacology (1, 11). We acknowledge the risk of type 1 statistical error given the number of SNPs that were evaluated, as well as two interventions and two colorectal polyp endpoints. Ideally, these findings would be replicated in an independent prospective cohort or intervention trial of aspirin use. However, given the number of tests conducted, we saw more significant associations than would be expected by chance, which suggests that our results are unlikely to be explained by chance. Moreover, the respective *q* values for associations between SNPs and colorectal polyp prevention efficacy of aspirin suggest that we report true positive findings. In addition, the power of our study was limited for rare homozygote SNPs, for which the minor allele frequency was small (<0.3), if the effect size is only moderate (IRR > 0.6). We also draw attention to the fact that the seAFOod trial population consisted of individuals who had undergone BCSP colonoscopy after a positive FOBt, thereby generating a predominantly male and white ethnicity cohort (17), which limits generalizability to other populations.

In summary, we report novel gene–chemoprevention (aspirin) interactions from the seAFOod polyp prevention trial. SNPs in COX-1, COX-2, LOX isoforms, and TP53 should be further evaluated as biomarkers of aspirin chemoprevention efficacy, alone and in combination with other polymorphisms reported to predict colorectal polyp and/or colorectal cancer risk reduction by aspirin (3).

## Supplementary Material

Supplementary Table 1 Supplementary Table 1 shows the list of SNPs that were analysed by the Fluidigm assay.

Supplementary Table 2 Supplementary Table 2 shows the results of Negative binomial regression analysis of total colorectal polyp number according to aspirin use stratified for each SNP

Supplementary Figure 1 Supplementary Figure 1 shows the distribution of individual total (combined adenomatous and serrated polyp) colorectal polyp counts according to seAFOod trial treatment allocation by factorial margins and PTGS SNP genotypes.

Supplementary Figure 2 Supplementary Figure 2 shows the distribution of individual total (combined adenomatous and serrated polyp) colorectal polyp counts according to seAFOod trial treatment allocation by factorial margins and ALOX SNP genotypes.

Supplementary Figure 3 Supplementary Figure 3 shows the distribution of individual total (combined adenomatous and serrated polyp) colorectal polyp counts according to seAFOod trial treatment allocation by factorial margins and TP53 rs104522 genotype.
