# Supplementary material for: Polymorphisms in Cyclooxygenase, Lipoxygenase, and TP53 Genes Predict Colorectal Polyp Risk Reduction by Aspirin in the seAFOod Polyp Prevention Trial
Source: Cancer Prev Res (Phila). 2023 Sep 26;16(11):621–9. doi: 10.1158/1940-6207.CAPR-23-0111 (PMC10618644; doi:10.1158/1940-6207.CAPR-23-0111)
Supplement: Supplementary Table 1 — shows the list of SNPs that were analysed by the Fluidigm assay. [file capr-23-0111_supplementary_table_1_suppst1.docx]

**Supplementary Table 1: List of SNPs characterised by the Fluidigm 96.96 IFC assay**

| dbSNP rs number | Fluidigm ID number | Minor Allele Frequency^1^ |
| --- | --- | --- |
| **Oxylipin synthesis and degradation** | | |
| ***PTGS-1* (COX-1) n=7** | | |
| rs4837960 | GTA0279715 | 0.139 |
| rs1330344 | GTA0192237 | 0.213 |
| rs10306114 | GTA0192222 | 0.066 |
| rs10306135 | GTA0279726 | 0.168 |
| rs10306164 | GTA0279710 | 0.179 |
| rs12551233 | GTA0204163 | 0.064 |
| rs10306194 | GTA0279853 | 0.149 |
| rs3842798 | GTA0204157 | 0.201 |
| rs4837963 | GTA0279848 | 0.078 |
| ***PTGS-2* (COX-2) n=5** | | |
| rs10911902 | GTA0192236 | 0.181 |
| rs2206593 | GTA0192225 | 0.063 |
| rs5275 | GTA0279868 | 0.337 |
| rs20417 | GTA0001420 | 0.073 |
| rs2745557 | GTA0279733 | 0.183 |
| rs4648310 | GTA0279706 | 0.021 |
| rs689466 | GTA0087714 | 0.179 |
| rs4648307 | GTA0279877 | 0.066 |
| ***ALOX5* (5-LOX) n=8** | | |
| rs12762303 | GTA0178432 | 0.161 |
| rs41526545 | GTA0279707 | 0.173 |
| rs7917687 | GTA0279864 | 0.047 |
| rs2115819 | GTA0153447 | 0.445 |
| rs11239516 | GTA0279863 | 0.098 |
| rs12264801 | GTA0279713 | 0.462 |
| rs2099171 | GTA0279721 | 0.294 |
| rs7090328 | GTA0279719 | 0.282 |
| rs12247163 | GTA0279732 | 0.201 |
| **ALOX12 (12-LOX) n=10** | | |
| rs2073438 | GTA0279697 | 0.277 |
| rs2292350 | GTA0279727 | 0.428 |
| rs1126667 | GTA0279728 | 0.420 |
| rs11078659 | GTA0279871 | 0.500 |
| rs11571339 | GTA0279724 | 0.082 |
| rs2920421 | GTA0155688 | 0.317 |
| rs312462 | GTA0279738 | 0.101 |
| rs2271316 | GTA0279855 | 0.493 |
| rs7338 | GTA0279722 | 0.138 |
| rs41283391 | GTA0279696 | 0.068 |
| rs11571364 | GTA0279701 | 0.062 |
| ***ALOX15* (15-LOX) n=3** | | |
| rs11568131 | GTA0279694 | 0.156 |
| rs2664593 | GTA0279709 | 0.207 |
| rs748694 | GTA0279699 | 0.472 |
| rs2619112 | GTA0279878 | 0.471 |
| rs11078527 | GTA0279703 | 0.149 |
| rs2255888 | GTA0279737 | 0.235 |
| ***ALOX5AP* n=10** | | |
| rs17074898 | GTA0279725 | 0.046 |
| rs12560847 | GTA0279736 | 0.293 |
| rs9578195 | GTA0279698 | 0.083 |
| rs12430915 | GTA0279718 | 0.075 |
| rs3885907 | GTA0084968 | 0.447 |
| rs3803277 | GTA0279730 | 0.447 |
| rs9506352 | GTA0279735 | 0.315 |
| rs4769874 | GTA0279723 | 0.044 |
| rs9579649 | GTA0279731 | 0.112 |
| rs9315050 | GTA0279716 | 0.076 |
| rs17216473 | GTA0279854 | 0.073 |
| ***15-PGDH* n=2** | | |
| rs45567139 | GTA0281593 | 0.057 |
| rs2555639 | GTA0281592 | 0.350 |
| **Aspirin ‘modifiers’ of CRC risk** | | |
| rs6983267 | GTA0040668 | 0.487 |
| rs2965667 | GTA0127016 | 0.027 |
| rs10505806 | GTA0279717 | 0.030 |
| rs16973225 | GTA0279705 | 0.046 |
| rs1042522 (TP53) | GTA0058417 | 0.263 |
| rs1109859 (PEMT) | GTA0263999 | 0.184 |
| rs4246215 (FEN1) | GTA0078104 | 0.278 |
| **Homocysteine metabolism** | | |
| rs1801133 (MTHFR) | GTA0005937 | 0.349 |
| rs1801131 (MTHFR) | GTA0005958 | 0.313 |
| rs4846052 (MTHFR) | GTA0279852 | 0.415 |
| **Polyunsaturated fatty acid metabolism** | | |
| ***FADS1* n=2** | | |
| rs174546 | GTA0274566 | 0.325 |
| rs174556 | GTA0192077 | 0.296 |
| ***FADS2* n=10** | | |
| rs77930731 | GTA0279702 | Not known |
| rs174570 | GTA0120100 | 0.126 |
| rs2072114 | GTA0192068 | 0.127 |
| rs174589 | GTA0279873 | 0.128 |
| rs2851682 | GTA0279729 | 0.087 |
| rs174602 | GTA0205371 | 0.201 |
| rs498793 | GTA0279720 | 0.406 |
| rs174611 | GTA0192058 | 0.286 |
| rs482548 | GTA0279734 | 0.096 |
| rs76497692 | GTA0279711 | 0.000 (0.03 African) |
| rs138766446 | GTA0279695 | 0.080 |
| rs968567 | GTA0279704 | 0.163 |
| rs174627 | GTA0192069 | 0.143 |
| ***FADS3* n=3** | | |
| rs174450 | GTA0279712 | 0.466 |
| rs1000778 | GTA0279706 | 0.258 |
| rs174455 | GTA0279866 | 0.360 |
| ***ELOVL2* n=5** | | |
| rs3734398 | GTA0279708 | 0.434 |
| rs2236212 | GTA0189358 | 0.430 |
| rs953413 | GTA0279872 | 0.418 |
| rs3798713 | GTA0279700 | 0.436 |
| rs9393903 | GTA0205367 | 0.239 |
| ***ELOVL5* n=1** | | |
| rs2397142 | GTA0279714 | 0.287 |
| ***APOE* n=2** | | |
| rs7412 | GTA0105056 | 0.083 |
| rs429358 | GTA0049268 | 0.068 |

Underline signifies that the SNP did not satisfy Hardy-Weinberg equilibrium (P<0.05 with Benjamini-Hochberg correction). Grey shade signifies that the SNP failed QC and was omitted from analysis. The Fluidigm ID number is the respective Fluidigm Assay ID for each SNP primer pair. ^1^Minor Allele Frequency is based on ALFA European data.
