# Supplementary material for: Polymorphisms in Cyclooxygenase, Lipoxygenase, and TP53 Genes Predict Colorectal Polyp Risk Reduction by Aspirin in the seAFOod Polyp Prevention Trial
Source: Cancer Prev Res (Phila). 2023 Sep 26;16(11):621–9. doi: 10.1158/1940-6207.CAPR-23-0111 (PMC10618644; doi:10.1158/1940-6207.CAPR-23-0111)
Supplement: Supplementary Figure 1 — shows the distribution of individual total (combined adenomatous and serrated polyp) colorectal polyp counts according to seAFOod trial treatment allocation by factorial margins and PTGS SNP genotypes. [file capr-23-0111_supplementary_figure_1_suppsf1.pptx]

## Slide 1
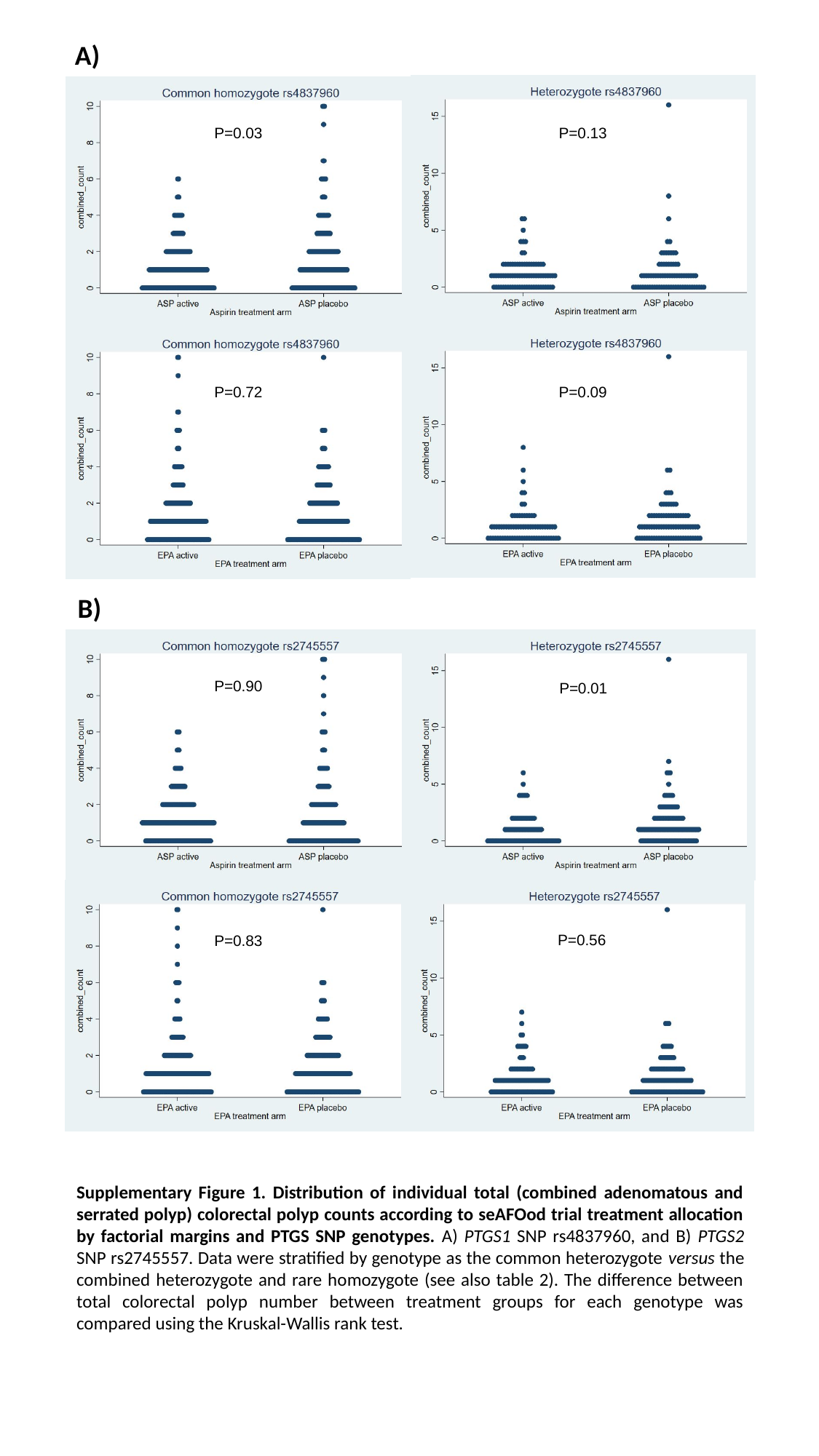

A)
P=0.03
P=0.13
P=0.72
P=0.09
B)
P=0.90
P=0.01
P=0.56
P=0.83
Supplementary Figure 1. Distribution of individual total (combined adenomatous and serrated polyp) colorectal polyp counts according to seAFOod trial treatment allocation by factorial margins and PTGS SNP genotypes. A) PTGS1 SNP rs4837960, and B) PTGS2 SNP rs2745557. Data were stratified by genotype as the common heterozygote versus the combined heterozygote and rare homozygote (see also table 2). The difference between total colorectal polyp number between treatment groups for each genotype was compared using the Kruskal-Wallis rank test.
